# Genetic correction improves prediction efficiency of serum tumor biomarkers on digestive cancer risk in the elderly Chinese cohort study

**DOI:** 10.18632/oncotarget.23205

**Published:** 2017-12-13

**Authors:** Ke Wang, Yansen Bai, Shi Chen, Jiao Huang, Jing Yuan, Weihong Chen, Ping Yao, Xiaoping Miao, Youjie Wang, Yuan Liang, Xiaomin Zhang, Meian He, Handong Yang, Qingyi Wei, Huan Guo, Sheng Wei

**Affiliations:** ^1^ Department of Epidemiology and Biostatistics, Ministry of Education Key Laboratory of Environment and Health, School of Public Health, Tongji Medical college, Huazhong University of Science and Technology, Wuhan, Hubei, China; ^2^ Department of Occupational and Environmental Health, School of Public Health, Tongji Medical College, Huazhong University of Science and Technology, Wuhan, Hubei, China; ^3^ Department of Nutrition and Food Hygiene, School of Public Health, Tongji Medical College, Huazhong University of Science and Technology, Wuhan, Hubei, China; ^4^ Department of Maternal and Child Health, School of Public Health, Tongji Medical College, Huazhong University of Science and Technology, Wuhan, Hubei, China; ^5^ Department of Social Medicine and Health Management, School of Public Health, Tongji Medical College, Huazhong University of Science and Technology, Wuhan, Hubei, China; ^6^ Dongfeng Central Hospital, Dongfeng Motor Corporation and Hubei University of Medicine, Shiyan, Hubei, China; ^7^ Duke Cancer Institute, Duke University Medical Center, Durham, NC, USA

**Keywords:** tumor biomarker, single nucleotide polymorphisms, genetic correction, digestive cancer, prediction efficiency

## Abstract

Although serum tumor biomarkers alpha-fetoprotein (AFP), carbohydrate antigen 19–9 (CA19–9) and carcinoembryonic antigen (CEA) have been used in digestive cancer risk prediction, the prediction efficiency remains unsatisfactory. The aim of this study was to evaluate whether genetic correction could improve the efficiency of these biomarkers for prediction of digestive cancer risk. We conducted a prospective analysis in 9,808 healthy individuals based on a cohort study in the elderly Chinese population. The genotypes of reported single nucleotide polymorphisms (SNPs) associated with serum AFP, CA19–9 and CEA were used to estimate the genetic corrected levels of these markers. Unconditional logistic regression analysis was performed to evaluate the risk of digestive cancer. The Harrell’s C-statistic was used to evaluate the discriminative ability of the raw levels and genetic corrected levels of biomarkers on digestive cancer risk. Up to October 2013, a total of 172 individuals were newly diagnosed with digestive cancer. With the genetic correction, higher odds ratios (ORs) for digestive cancer risk were found for the genetic corrected levels of tumor biomarkers compared with their raw serum levels (1.57 vs. 1.65 for AFP; 1.19 vs. 1.21 for CA19–9; 1.09 vs. 1.10 for CEA, respectively). The same results were observed in the Harrell’s C-statistic analyses. Genetic correction improved the prediction efficiency of tumor biomarkers on the digestive cancer risk in an elderly Chinese population. Our findings provide evidence for further studies of genetic effects on tumor biomarker to improve the predictive efficiency on cancer risk.

## INTRODUCTION

Digestive cancer, including cancers of the liver, stomach, esophagus, colorectal and pancreas, is one type of the most frequent cancers in the world, particularly in China [1, 2]. The prediction efficacy for cancer risk plays a decisive role in digestive cancer prevention [3, 4]. Consequently, it is imperative to improve the prediction efficacy of digestive cancer risk. As the most widely used serum tumor markers, alpha-fetoprotein (AFP), carbohydrate antigen 19–9 (CA19–9) and carcinoembryonic antigen (CEA) have been in application since the 1970s [5, 6]. However, the predictive efficacy of these tumor biomarkers for digestive cancer has not been satisfactory. Serum levels of these biomarkers may be elevated due to underlying conditions other than digestive cancer, including non-digestive cancer and various benign diseases. Previous studies have shown that the efficiency in predicting digestive cancer based only on serum AFP remains poor [7, 8]. The same findings were reported for CA19–9 and CEA [9–11]. As a result, it is unsatisfactory to predict the risk of digestive cancer using serum levels of these tumor biomarkers.

Recent studies have found that the levels of tumor biomarkers can also be affected by hereditary variation, not only by other factors such as inflammation and malignant tumors [12–14]. For example, it was reported that the prediction accuracy could be improved with the genetic correction of PSA level than without correction in prostate cancer risk prediction, suggesting that correction for such genetic variants could provide a better individual estimate of serum PSA level [15]. Therefore, we proposed that prediction efficiency of digestive cancer risk using serum tumor biomarkers could be improved by combining genetic variations. Capitalized on our previously published genome-wide association study, we identified several single nucleotide polymorphisms (SNPs) to be associated with serum AFP, CA19–9 and CEA levels [16]. We hypothesized that these variants could account, in part, for the observed variability in assessing biomarker-related cancer risk among individuals. Therefore, genetic correction may improve the efficiency in predicting digestive cancer using serum levels of tumor biomarkers.

To address this hypothesis, we performed a prospective cohort study to evaluate the value of genetic correction of tumor biomarker for the prediction of digestive cancer risk in a large Chinese population. Our objective was to evaluate whether the genetic correction could improve the prediction efficiency of the raw levels of serum biomarkers on digestive cancer risk.

## RESULTS

### Baseline characteristics

A total of 172 participants were newly diagnosed with digestive cancer during follow-up (39 with liver cancer, 35 with gastric cancer, 64 with colorectal cancer, 20 with esophageal cancer and 14 with pancreatic cancer). The cumulative incidence rate of digestive cancer was 17.54 cases per 1,000 people. Baseline characteristics of all the individuals with or without digestive cancer, including demographic, lifestyle and biochemical indicators, are presented in Table 1. The mean (± SD) age was 65.26 (± 7.32) years, and BMI was 24.02 (± 3.25) kg/m^2^ for subjects with digestive cancer. No significant differences were found between the participants with and without digestive cancer for BMI, drinking status, physical activity or family history of cancer. Compared with the subjects without digestive cancer, more subjects with digestive cancer reported having a history of smoking (*P* = 0.027). Males and older people were more common among the individuals diagnosed with digestive cancer than those without (both *P* < 0.001).

**Table 1:** Baseline characteristics of participants with and without digestive cancer in the present study from the DFTJ cohort study (*n* = 9808)

Characteristic	With digestive cancer (*n* = 172)	Without digestive cancer (*n* = 9636)	*P*
Age (years)	65.26 ± 7.32	62.03 ± 7.78	< 0.001
Gender			< 0.001
Male	105 (61.0%)	4493 (46.6%)	
Female	67 (39.0%)	5143 (53.4%)	
BMI (kg/m^2^)	24.02 ± 3.25	24.34 ± 3.32	0.219
Smoking status			0.027
Ever	65 (38.0%)	2883 (30.2%)	
Never	106 (62.0%)	6669 (69.8%)	
Drinking status			0.159
Ever	55 (32.0%)	2609 (27.2%)	
Never	117 (68.0%)	6998 (72.8%)	
Physical activity			0.099
Yes	159 (92.4%)	8484 (88.4%)	
No	13 (7.56%)	1115 (11.6%)	
Family history of cancer			0.607
Yes	4 (2.3%)	289 (3.0%)	
No	168 (97.7%)	9347 (97.0%)	
AFP (ng/ml)	2.74 (1.33–3.91)	2.70 (0.96–3.90)	< 0.001
CA19–9 (u/ml)	8.82 (4.58–17.84)	7.79 (3.91–14.73)	0.008
CEA (ng/ml)	2.18 (1.26–3.80)	1.79 (1.06–2.74)	< 0.001
Genetic corrected AFP (ng/ml)	2.77 (1.28–3.83)	2.58 (0.85–3.72)	< 0.001
Genetic corrected CA19–9 (u/ml)	7.74 (3.77–14.84)	6.23 (3.03–11.63)	< 0.001
Genetic corrected CEA (ng/ml)	2.15 (1.23–3.97)	1.76 (1.06–2.69)	< 0.001

Some data are not consistent with total number because of missing (< 1.0%).

Data are presented as number (percentage) or means ± standard deviation for normally distributed continuous variables, median and interquartile range for continuous variables that are not normally distributed.

Abbreviations: DFTJ cohort: Dongfeng-Tongji cohort; BMI: body mass index; AFP: alpha-fetoprotein; CA19–9: carbohydrate antigen 19–9; CEA: carcinoembryonic antigen.

### Effect of SNPs on serum tumor biomarker level

The estimated relative genotype effect of SNPs associated with serum AFP, CA19–9 and CEA levels are shown in Table 2. As a result, there was a significant decrease in the total AFP level after genetic correction (*P* < 0.001). After adjustment, the total level of AFP were 8.8% lower than the AFP level prior to adjustment (7.17 ng/ml for corrected AFP level compared with 7.86 ng/ml for measured AFP level). Inversely, serum level of AFP after genetic correction were noticeably and significantly higher in subjects diagnosed with digestive cancer compared to those who were free of digestive cancer (7.93 ng/ml vs. 2.73 ng/ml, *P* < 0.001). Similar results for the serum level of CA19–9 and CEA were also presented in Table 1. The distribution of each serum biomarker level before and after genetic correction in differential types of digestive cancer is shown in Supplementary Figures 1–2.

**Table 2:** Estimates on the relative genotype effect for SNPs associated with the tumor biomarker levels of AFP, CA19–9 and CEA

SNP	Allele	Chr	Position (bp)	Allelic Frequency	Relative Allelic effect	XX effect	OX effect	OO effect
AFP								
rs12506899	T	4	74, 5 38, 147	0.33	1.08	1.10	1.02	0.95
rs2251844	T	15	41, 623, 770	0.47	1.10	0.92	1.00	1.11
CA19–9								
rs17271883	A	19	5, 785, 212	0.44	1.29	0.78	1.01	1.31
rs3760775	G	19	5, 792, 356	0.25	1.49	1.76	1.18	0.79
rs265548	T	19	17, 763, 334	0.23	1.04	1.07	1.02	0.98
rs1047781	A	19	53, 898, 443	0.40	1.38	0.76	1.04	1.43
CEA								
rs8176749	C	9	135, 121, 009	0.21	1.17	0.93	1.09	1.28
rs8176720	T	9	135, 122, 694	0.45	1.08	0.93	1.01	1.09
rs3670775	G	19	5, 792, 356	0.25	1.06	0.97	1.03	1.09
rs1047781	A	19	53, 898, 443	0.40	1.17	0.88	1.03	1.20
rs441810	A	21	41, 620, 777	0.18	1.01	1.00	1.01	1.02

Shown are the SNPs associated with AFP, CA19–9 and CEA levels and their alleles and the relative genotype effect on the levels of tumor biomarkers: for homozygous (XX), heterozygous (OX), and non-carriers (OO) of the allele associated with biomarkers levels.

Abbreviations: SNP: single nucleotide polymorphism; AFP: alpha-fetoprotein; CA19–9: carbohydrate antigen 19–9; CEA: carcinoembryonic antigen; Chr: Chromosome.

In addition, the analyses of associations between these SNPs and digestive cancer were performed and are presented Supplementary Table 1. The correlations were not statistical significant among all SNPs (all *P* > 0.05). The functional SNPs highly correlated with target SNPs associated with serum levels of AFP, CA19–9 and CEA are also shown in Supplementary Table 2.

### The association between genetic corrected tumor biomarker level and the risk of digestive cancer

The risk of digestive cancer associated with genetic corrected tumor biomarkers are shown in Table 3. The risk of digestive cancer elevated with the increase of serum biomarker level before and after genetic correction. With the genetic correction, a higher OR for digestive cancer was found when compared with the raw serum levels of AFP, CA19–9, CEA and their combination. After adjustment for age, sex, smoking status, drinking status, BMI and other covariates in different models, the associations were still statistically significant. The adjusted ORs for the risk of digestive cancer associated with raw levels of serum AFP, CA19–9 and CEA were 1.57 (1.18, 2.09), 1.19 (1.07, 1.33) and 1.09 (0.95, 1.25), respectively. The ORs of genetic corrected AFP, CA19–9 and CEA levels were 1.65 (1.22, 2.24), 1.21 (1.06, 1.38), 1.10 (0.96, 1.26). Among these genetic corrected biomarkers, the combination biomarker level was the strongest independent predictor of digestive cancer after adjustment for several covariates (OR = 3.01, 95% CI = 2.20–4.12). The subgroup analyses for the association between combination biomarker level and the risk of digestive cancer are also performed. The combination marker level with genetic correction were associated with the risk of digestive cancer in males, higher BMI, older groups and people having history of smoking and drinking. The details are presented in Supplementary Table 3.

**Table 3:** ORs and 95% CIs for the risk of incident digestive cancer based on baseline raw biomarkers levels and the levels after genetic correction in the elderly Chinese people

Biomarkers	Raw level		Genetic corrected level	
	OR (95% CI)	*P*	OR (95% CI)	*P*
AFP				
Model 1	1.61 (1.21, 2.14)	0.001	1.70 (1.25, 2.30)	< 0.001
Model 2	1.60 (1.20, 2.13)	0.001	1.68 (1.23, 2.28)	0.001
Model 3	1.57 (1.18, 2.09)	0.002	1.65 (1.22, 2.24)	0.001
CA19–9				
Model 1	1.19 (1.07, 1.33)	0.002	1.21 (1.06, 1.38)	0.005
Model 2	1.18 (1.06, 1.32)	0.003	1.20 (1.05, 1.37)	0.008
Model 3	1.19 (1.07, 1.33)	0.001	1.21 (1.06, 1.38)	0.004
CEA				
Model 1	1.10 (0.97, 1.26)	0.152	1.11 (0.97, 1.27)	0.138
Model 2	1.07 (0.92, 1.23)	0.383	1.08 (0.92, 1.24)	0.363
Model 3	1.09 (0.95, 1.25)	0.202	1.10 (0.96, 1.26)	0.187
AFP+CA19–9+CEA				
Model 1	1.33 (1.17, 1.51)	< 0.001	2.72 (2.01, 3.67)	< 0.001
Model 2	1.32 (1.16, 1.49)	< 0.001	2.50 (1.85, 3.37)	< 0.001
Model 3	1.51 (1.24, 1.85)	< 0.001	3.01 (2.20, 4.12)	< 0.001

Model 1, univariate model.

Model 2, adjusted for age, gender, smoking status, drinking status.

Model 3, adjusted for the variables in model 2 plus BMI, physical activity, family history of cancer.

Abbreviations: AFP: alpha-fetoprotein; CA19–9: carbohydrate antigen 19–9; CEA: carcinoembryonic antigen; OR: odds ratio; 95% CI: 95% confidence intervals; BMI: body mass index.

### The Harrell’s C-statistic analyses for the discriminatory ability of digestive cancer risk

To estimate the discriminatory ability for the serum biomarker level and the genetic corrected level on the risk of digestive cancer, the C-statistic value was calculated as shown in Table 4. After genetic correction, the C-statistic for AF*P* values was higher (0.529, 95% CI, 0.485–0.573) than that with raw values (0.521, 95% CI, 0.477–0.564; *P* < 0.001), the similar results for values of CA19–9, CEA, and their combination were seen in Table 4. In the differential prediction markers, the combination with genetic correction exhibited a high discriminatory ability for digestive cancer (C-statistic = 0.649, 95% CI, 0.608–0.690) compared with the combination with raw levels of tumor biomarkers (C-statistic = 0.531, 95% CI, 0.486–0.575; *P* < 0.001). The analysis of each tumor biomarker and in combination for the discrimination of each digestive cancer were presented in Supplementary Tables 4–7.

**Table 4:** The Harrell’s C-statistics of baseline raw levels and the levels of AFP, CA19–9 and CEA after genetic correction for the discrimination of digestive cancer

Biomarkers	Harrell’s C-statistic	95% CI	*P*^a^
AFP			
Raw level	0.521	0.477–0.564	
Genetic corrected level	0.529	0.485–0.573	< 0.001
CA19–9			
Raw level	0.555	0.509–0.602	
Genetic corrected level	0.560	0.514–0.605	0.557
CEA			
Raw level	0.580	0.532–0.628	
Genetic corrected level	0.581	0.532–0.629	0.966
AFP + CA19–9 + CEA			
Raw level	0.531	0.486–0.575	
Genetic corrected level	0.649	0.608–0.690	< 0.001

^a^ The C-statistic for the level of each tumor biomarker after genetic correction compared with the C-statistic for the raw level.

Abbreviations: AFP: alpha-fetoprotein; CA19–9: carbohydrate antigen 19–9; CEA: carcinoembryonic antigen; 95% CI: 95% confidence intervals.

## DISCUSSION

To the best of our knowledge, the current study is the first time to evaluate the discriminative ability of the genetic corrected AFP, CA19–9 and CEA levels on digestive cancer risk in a prospective cohort study. Our findings demonstrate that the genetic correction of these tumor biomarkers levels could improve the prediction efficiency of raw biomarker level for digestive cancer risk in the Chinese population. For the Harrell’s C-statistic analyses, with the efficacy of genetic correction, the C-statistic values were higher compared with the raw levels of AFP, CA19–9 and CEA. The current study suggests that the discriminatory ability of tumor markers on digestive cancer risk could be improved by genetic correction on the raw serum levels in elderly Chinese.

Tumor biomarkers included AFP, CA19–9 and CEA are commonly used biomarkers for cancer prognosis and therapy, while it is not satisfactory in terms of cancer prediction because of the poor prediction efficiency [17–19]. However, our study found that genetic correction could improve the prediction efficiency of biomarkers for digestive cancer risk. In consideration that digestive cancer is a kind of deadly cancer with poor early detection, on the other hand, genetic variation is intimately linked in the development of digestive cancer, there is a clear need for personalized prediction early on digestive cancer risk based on individual genomic information to improve the efficacy of prediction performance [20]. With this background finally at hands, it is important to emphasize that the advances in human genetics studies will lead to advances in cancer prediction. We are on the cusp of a great and exciting change in personalized care. The biological mechanisms for the association between these SNPs and tumor biomarkers are shown as follows.

AFP is a single-chain oncofetal glycoprotein tumor antigen, the serum level of AFP indicates a growth-regulatory activity toward developing digestive cancer [21]. The SNPs rs12506899 in the gene *AFP* and rs2251844 in *HISPPD2A* were found to be associated with serum level of AFP in our previous GWAS study [16]. These SNPs may have effect on the concentrations of AFP directly or indirectly. The posttranscriptional changes in *AFP* expression could influence the concentration of serum AFP through α-fetoprotein regulator I which can regulate *AFP* repression with the effect of *AFP* promoter [22, 23]. rs12506899 is located at intron of *AFP*, but it had a high link with rs6834059 (LD = 0.76 in Chinese) which can affect transcription factor binding site of *AFP* to regulate the expression of *AFP*. In addition, we found that there was no association between these SNPs and digestive cancer risk in the current study. This result suggested that rs12506899 may affect the concentrations of AFP through the relationship between rs12506899 and rs6834059. A similar situation was seen in rs2251844 on expression of *HISPPD2A*.

CA19–9 is a carbohydrate antigen as an important biomarker for digestive cancer [24]. Several SNPs including rs17271883, rs3760775 in *FUT6*, rs265548 in *B3GNT3* and rs1047781 in *FUT2* were found to be associated with CA19–9 level in Chinese [16]. The *B3GNT3* located at 19p13.1 encodes one of the members in the β-1,3-N-acetylglucosmaniyl transferase family as the major constitution of dimeric sialyl, which is the carbohydrate antigenic epitope of CA19–9 [25]. SNP Function prediction analyses demonstrated that rs265548 is located at a transcription factor binding site of *B3GNT3* suggesting rs265548 may have impact on the transcription level of target gene and further regulate the individual level of serum CA19–9. Similarly, rs1047781is a transcript variant in *FUT2*, and some studies reported that *FUT2* could down-regulate the serum concentration of CA19–9 by involving in the synthesis of CA19–9 [26]. Therefore, it is biologically reasonable that SNPs could influence the concentrations of CA19–9 though these mechanisms. The functional relationship between SNPs in *FUT6* and CA19–9 level remains to be explored.

CEA has been used as a tumor biomarker for a long period of time [27]. The SNPs rs8176749 and rs8176720 in the gene *ABO*, rs3670775 in *FUT6*, rs1047781 in *FUT2* and rs441810 in *FAM3B* were found to be associated with serum CEA level [16]. Although the rs8176749 and rs8176720 in *ABO* both are synonymous variants which have not change amino acid sequences, but rs8176749 has a high linkage disequilibrium with rs8176751 (LD = 0.97) which locates at a microRNA-binding site in *ABO* and may influence the expression of *ABO*. And same glycoprotein carrier molecules were shared by blood A, B antigens and CEA [28]. That may explain the relation of *ABO* gene expression regulation to CEA level. The biological mechanisms of SNP rs 8176720 on CEA leval are not clear now. *FUT6* encodes the fucosyltransferases to facilitate the construction of Lewis antigens, which have the same carrier molecule as CEA [29]. The rs3670775 locates at the promoter region of *FUT6* which may affect the expression of *FUT6*. The rs1047781is a non-synonymous variant in *FUT2* which could transform isoleucine into phenylalanine with the change from A to T allele [30]. These may be the biological mechanisms for the association between these SNPs and the CEA serum level. The molecular mechanism for the association between rs441810 in *FAM3B* and CEA concentrations is still not clear.

Our study suggested that genetic variants play an important role in personal serum biomarker level. The level of tumor biomarker with genetic correction could reflect the individualized biomarker level more objectively compared to the raw level of tumor biomarker. Such genetic corrected individual level of tumor biomarker may improve its performance to predict the risk of digestive cancer. However, several limitations of the current study should be addressed. First, we only considered SNPs associated with serum tumor biomarker level for genetic correction. In fact, there may be more genetic variants associated with serum tumor marker level besides SNPs. Considering genetic variants other than SNPs in the future will help us to learn more about the role of genetic corrected biomarkers on cancer risk prediction. Second, only elderly people were enrolled in the present study, and thus we may not be able to extrapolate the utility of genetic correction biomarker level to the young population. However, all participants in the current study were former employees of the Dongfeng automobile enterprise and included people from the local and almost all other areas of China. The subjects in the current study have a certain representativeness. Although there are some limitations in the current study, our findings still show the prediction value of genetic corrected levels of serum tumor biomarkers on digestive cancer risk. Further studies, including a longer follow-up period, need to be performed, which will help clarify the utility of these serum biomarkers as predictive indicators of clinically relevant endpoints.

In conclusion, we have found that the prediction efficiency of serum AFP, CA19–9 and CEA levels on digestive cancer risk could be improved by taking the effects of genetic correction into account. Our findings suggest that it may be necessary to consider the impact of individual genetic variation on these biomarkers for cancer risk prediction in the future.

## MATERIALS AND METHODS

### Study population

The current study was based on the Dongfeng-Tongji cohort (DFTJ cohort), which was launched in 2008. The profile of the DFTJ cohort has been described elsewhere [31]. In brief, the DFTJ cohort consists of 27,009 people who had retired in 2008 from a Dongfeng automobile enterprise in Hubei, China. After they provided a signed informed consent, all participants started up from September 2008 to June 2010 by collecting detailed information about questionnaire data, clinical examination data and laboratory data at the baseline, and were followed up until October 2013.

At the meaning time, several SNPs were found to be associated with serum level of AFP (rs12506899 in *AFP* and rs2251844 in *HISPPD2A*), CA19–9 (rs17271883 in *FUT6*, rs3760775 in *FUT6*, rs265548 in *B3GNT3*, rs1047781 in *FUT2*), and CEA concentrations (rs8176749, rs8176720 and rs8176672 in *ABO*, rs3670775, rs1047781, rs441810 in *FAM3B*) in a genome-wide association study [16]. Among these SNPs, the SNP rs8176672 was in high linkage disequilibrium with rs8176749 (LD = 1) in CHB population, and finally rs8176749 was selected in this study for its lower *P* value (2.89 × 10^–9^ vs. 2.28 × 10^–2^) in previous study. As a result, a total of 10,197 healthy individuals without cancer and other chronic diseases diagnosis before from the DFTJ cohort study (including subjects in the GWAS discovery stage and validation stage) with genotyping information of those SNPs associated with tumor biomarkers were included in the current study.

The population of this study was based on our previous genome-wide association study from the DFTJ cohort (*n* = 10,197). The 389 participants unable to be contacted within the follow-up were excluded (*n* = 389). In total, 9,808 subjects meeting the criteria were included in the current study.

### Testing of blood AFP, CA19–9 and CEA concentrations

All study participants received physical examinations at Dongfeng Central Hospital after fasting overnight until the following morning with trained doctors and technicians at the baseline. Dongfeng Central Hospital is a main medical center for DMC retired employees as a large tertiary general hospital. The laboratory of Dongfeng Central Hospital was certified by Clinical Laboratory Center of the Ministry of Health in China. Fifteen milliliters of fasting blood was collected in coagulation tubes from each participant at the hospital’s laboratory of Dongfeng Central Hospital. Blood serum levels of AFP, CA19–9 and CEA were measured by immunoassay with Architect Ci8200 automatic analyzer (Abbott Park, Abbott Laboratories, USA). All tests were performed and interpreted by experienced and trained staff. When the concentration of tumor biomarkers was less than 0.01 ng/ml (u/ml) (the lowest limit of the standard curve), the value was given a ‘low’ value of 0.005 ng/ml (u/ml).

### The follow-up and the diagnosis of digestive cancer

All the participants in the DFTJ cohort study were followed up through a unique medical insurance number from Dongfeng Medical Insurance Center, thus facilitating the physical examination and questionnaire interview as well as reports of disease status and causes of deaths. All medical records of the participants were provided by Dongfeng Medical Insurance Center and the Dongfeng Central Hospital. The diagnosis of major diseases was verified by reviewing the medical records in the follow-up database. The follow-up of the participants in the DFTJ cohort was until October 2013.

The new diagnosis of primary digestive cancer in the current study was based on the following criteria: (1) histological and pathological diagnostic criteria of the WHO (including cancers of the liver, stomach, esophagus, colorectal and pancreas), and (2) the patient had not undergone preoperative anti-cancer treatment and there was no incidence of extrahepatic metastases prior to diagnosis [32–35].

### Statistical analysis

The information about demographic, biochemical and histological data was summarized and tabulated. The characteristics of the participants were presented as n (%) or means ± standard deviation (mean ± SD) for normally distributed continuous variables, medians and interquartile range for continuous variables that were not normally distributed. For numerical variables, the Student’s *t*-test was used to assess the significance of differences. For comparisons between quantitative variables, the Mann-Whitney U test was used. The Pearson Chi-squared test was used for comparative purposes between categorical variables. Unconditional logistic regression analysis was performed to evaluate the risk of digestive cancer in multivariate analyses with different models. The odds ratios (ORs) and their 95% confidence intervals (CIs) were presented together.

The serum levels of tumor biomarkers after genetic correction were estimated by dividing the measured biomarker level and the predicted combined genetic effect. A classical linear regression was used for each SNP with the genotype as an independent variable, and the level of each tumor biomarker as a response variable to calculate the association. In addition, a log-transformed analysis of each biomarker was performed for testing the standardized value, which was then back-transformed to evaluate effect of each genotype. The combined genetic effect of all SNPs was calculated by multiplying the genotypic effect of each SNP with a multiplicative model [15].

To assess the discriminative ability for digestive cancer, the Harrell’s C-statistic from a Cox proportional hazards regression model was generated for the tumor biomarkers and their combination [36, 37]. The C-statistic value with 95% CI was then calculated for the serum level of each tumor biomarker with and without genetic correction. All two-sided *P* values less than 0.05 were considered statistically significant. The box-plot graphs were created using GraphPad Prism 5 software. All statistical analyses were performed using SAS version 9.4 (SAS Institute, Cary, NC, USA) and Empower Stats (http://www.empowerstats.com).

### Ethical approval

All procedures performed in studies involving human participants and experiments were in accordance with the ethical standards of the institutional and/or national research committee and with the 1964 Helsinki declaration and its later amendments or comparable ethical standards. The human experimental protocols in this study were approved by the Medical Ethics Committee of Dongfeng General Hospital and the School of Public Health, Tongji Medical College. The investigators obtained written informed consent from each participant.

## SUPPLEMENTARY MATERIALS FIGURES AND TABLES


