# Supplementary material for: Genetic correction improves prediction efficiency of serum tumor biomarkers on digestive cancer risk in the elderly Chinese cohort study
Source: Oncotarget. 2017 Dec 13;9(7):7389–97. doi: 10.18632/oncotarget.23205 (PMC5800910; doi:10.18632/oncotarget.23205)
Supplement: Supplementary file 1 [file oncotarget-09-7389-s001.pdf]

Genetic correction improves prediction efficiency of serum tumor biomarkers on digestive cancer risk in the elderly chinese cohort study

SUPPLEMENTARY MATERIALS

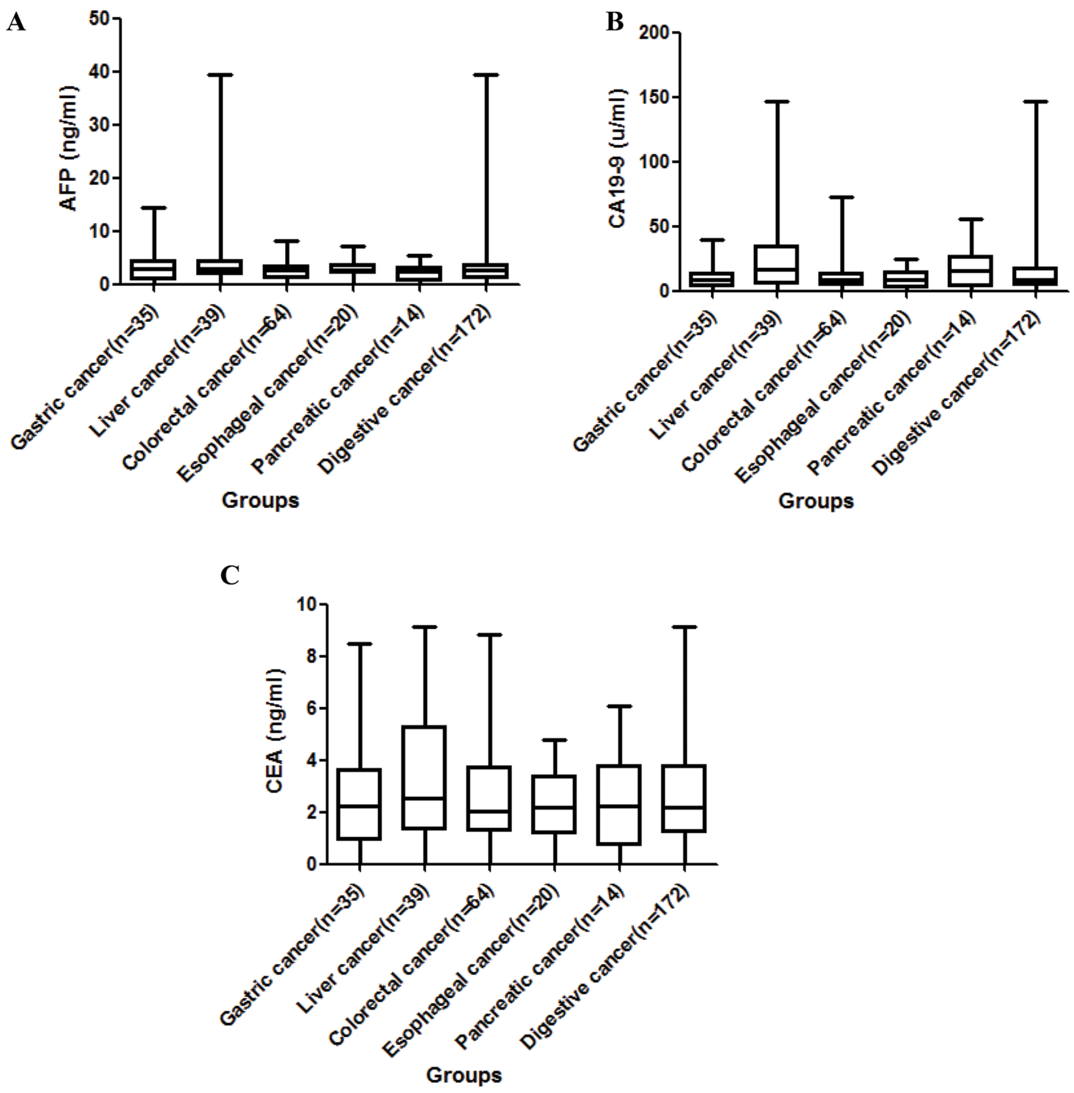

Supplementary Figure 1: Distribution of the raw levels of AFP, CA19–9 and CEA in the differential types of digestive cancer.

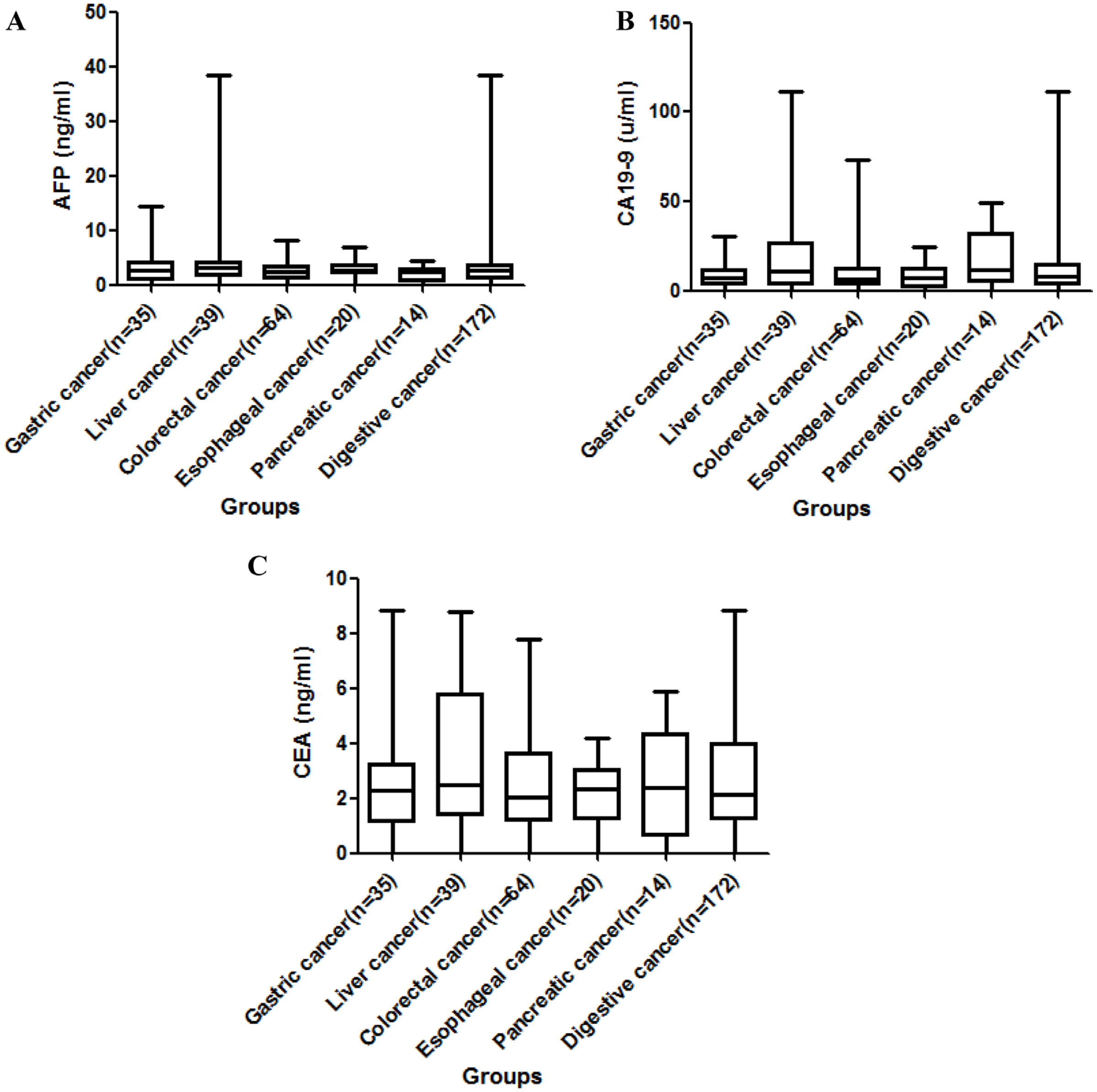

Supplementary Figure 2: Distribution of AFP, CA19–9 and CEA levels after genetic correction in the differential types of digestive cancer.

Supplementary Table 1: The association analyses between SNPs and digestive cancer risk

SNP	OR[a]	95% CI	*P*
rs12506899	1.20	0.93–1.56	0.160
rs2251844	1.09	0.84–1.40	0.522
rs17271883	1.07	0.78–1.46	0.681
rs3760775	1.26	0.89–1.78	0.186
rs265548	1.05	0.80–1.37	0.747
rs1047781	1.14	0.89–1.45	0.305
rs8176749	1.04	0.78–1.39	0.785
rs8176720	0.87	0.69–1.11	0.271
rs441810	0.97	0.70–1.34	0.836

[a]Adjusted for age, gender, smoking status, drinking status, BMI, physical activity, family history of cancer.
Abbreviations: SNP: single nucleotide polymorphism; OR: odds ratio; 95%CI: 95% confidence intervals; BMI: body mass index.

Supplementary Table 2: The functional SNPs highly linked with target SNPs associated with AFP, CA19–9, CEA levels and their potential functions

Target SNPs	SNP function	High correlated functional SNPs[a]	
		TFBS	miRanda
rs12506899	intron variant	rs6834059, rs13124940, rs13138734, rs3796676, rs4403016, rs4446279	-
rs2251844	intron variant	rs1869258, rs506120, rs529611	rs3087657, rs572837, rs689797
rs17271883	intron variant	rs3760774	-
rs3760775	upstream variant	-	-
rs265548	TFBS	rs265545, rs36692	-
rs1047781	transcript variant	-	-
rs8176749	synonymous codon	-	rs8176751
rs8176720	synonymous codon	-	-
rs441810	intron variant	-	-

[a]The information was obtained from http://snpinfo.niehs.nih.gov/.
Abbreviations: SNP: single nucleotide polymorphism; AFP: alpha-fetoprotein; CA19–9: carbohydrate antigen 19–9; CEA: carcinoembryonic antigen; TFBS: Transcription Factor Binding Sites; miRanda: MicroRNA-binding sites.

Supplementary Table 3: ORs (95% CIs) for the risk of digestive cancer based on combination marker level after genetic correction, by subgroups

Subgroups	OR	95% CI	*P*
Gender[a]			
Male	2.91	2.00–4.23	< 0.001
Female	1.95	0.90–4.24	0.090
Age (years)[b]			
< 62	1.92	0.83–4.40	0.126
≥ 62	3.00	2.08–4.33	< 0.01
BMI (kg/m^2)[b]			
< 24	2.62	1.69–4.05	< 0.001
≥ 24	2.86	1.75–4.67	< 0.001
Smoking status[b]			
Ever	2.85	1.93–4.20	< 0.001
Never	2.38	1.30–4.35	0.005
Drinking status[b]			
Ever	4.13	2.04–8.37	< 0.001
Never	2.49	1.73–3.58	< 0.001

[a]Adjusted for age, smoking status, drinking status, BMI, physical activity, family history of cancer.
[b]Adjusted for the other variables.
Abbreviations: OR: odds ratio; 95% CI: 95% confidence intervals; BMI: body mass index.

Supplementary Table 4: The Harrell's C-statistic analyses of baseline raw level and genetic corrected level of AFP for the discrimination of each digestive cancer

Digestive cancer	Harrell's C-statistic	95%CI	*P*[a]
**Gastric cancer**			
AFP level	0.522	0.419–0.625	
Genetic corrected AFP level	0.531	0.428–0.634	0.116
**Liver cancer**			
AFP level	0.603	0.504–0.702	
Genetic corrected AFP level	0.613	0.514–0.712	0.077
**Colorectal cancer**			
AFP level	0.523	0.456–0.591	
Genetic corrected AFP level	0.524	0.458–0.591	0.032
**Esophageal cancer**			
AFP level	0.550	0.437–0.662	
Genetic corrected AFP level	0.565	0.454–0.675	0.026
**Pancreatic cancer**			
AFP level	0.559	0.426–0.693	
Genetic corrected AFP level	0.560	0.431–0.689	0.944

[a]The C-statistic for genetic corrected level of AFP compared with the C-statistic for the raw level.
Abbreviations: AFP: alpha-fetoprotein; 95% CI: 95% confidence intervals.

Supplementary Table 5: The Harrell's C-statistic analyses of baseline raw level and genetic corrected level of CA19–9 for the discrimination of each digestive cancer

Digestive cancer	Harrell's C-statistic	95% CI	P[a]
**Gastric cancer**			
CA19-9 level	0.508	0.415–0.601	0.034
Genetic corrected CA19–9 level	0.543	0.457–0.629	
**Liver cancer**			
CA19–9 level	0.620	0.512–0.727	0.638
Genetic corrected CA19–9 level	0.640	0.537–0.723	
**Colorectal cancer**			
CA19–9 level	0.524	0.451–0.597	0.580
Genetic corrected CA19–9 level	0.525	0.452–0.597	
**Esophageal cancer**			
CA19–9 level	0.500	0.373–0.615	0.446
Gene–corrected CA19–9 level	0.507	0.379–0.636	
**Pancreatic cancer**			
CA19–9 level	0.638	0.448–0.828	0.935
Genetic corrected CA19–9 level	0.640	0.458–0.823	

[a]The C-statistic for genetic corrected level of CA19–9 compared with the C-statistic for the raw level.
Abbreviations: CA19–9: carbohydrate antigen 19–9; 95% CI: 95% confidence intervals.

Supplementary Table 6: The Harrell's C-statistic analyses of baseline raw level and genetic corrected level of CEA for the discrimination of each digestive cancer

Digestive cancer	Harrell's C-statistic	95% CI	P^{a}
**Gastric cancer**			
CEA level	0.538	0.430–0.646	
Genetic corrected CEA level	0.545	0.435–0.655	0.559
**Liver cancer**			
CEA level	0.629	0.522–0.736	
Genetic corrected CEA level	0.637	0.530–0.744	0.394
**Colorectal cancer**			
CEA level	0.568	0.492–0.645	
Genetic corrected CEA level	0.572	0.495–0.649	0.676
**Esophageal cancer**			
CEA level	0.558	0.432–0.685	
Genetic corrected CEA level	0.558	0.435–0.681	0.914
**Pancreatic cancer**			
CEA level	0.560	0.379–0.741	
Genetic corrected CEA level	0.579	0.387–0.771	0.219

[a]The C-statistic for genetic corrected level of CEA compared with the C-statistic for the raw level.
Abbreviations: CEA: carcinoembryonic antigen; 95% CI: 95% confidence intervals.

Supplementary Table 7: The Harrell's C-statistic analyses of baseline raw level and genetic corrected level of combination marker for the discrimination of each digestive cancer

Digestive cancer	Harrell's C-statistic	95% CI	P^a
**Gastric cancer**			
AFP + CA19–9 + CEA	0.539	0.435–0.643	0.880
Genetic corrected level	0.546	0.441–0.651	
**Liver cancer**			
AFP + CA19–9 + CEA	0.620	0.520–0.721	0.030
Genetic corrected level	0.704	0.599–0.809	
**Colorectal cancer**			
AFP + CA19–9 + CEA	0.509	0.439–0.579	0.075
Genetic corrected level	0.564	0.487–0.641	
**Esophageal cancer**			
AFP + CA19–9 + CEA	0.555	0.439–0.670	0.976
Genetic corrected level	0.556	0.443–0.669	
**Pancreatic cancer**			
AFP + CA19–9 + CEA	0.500	0.317–0.587	0.953
Genetic corrected level	0.598	0.411–0.784	

[a]The C-statistic for genetic corrected level of combination marker compared with the C-statistic for the raw level.
Abbreviations: AFP: alpha-fetoprotein; CA19–9: carbohydrate antigen 19–9; CEA: carcinoembryonic antigen; 95% CI: 95% confidence intervals.
